# Antidromic Analog Signaling

**DOI:** 10.3389/fncel.2019.00354

**Published:** 2019-08-02

**Authors:** Federico F. Trigo

**Affiliations:** ^1^CNRS UMR8003, SPPIN Laboratory, Cerebellar Neurophysiology Group, Faculté des Sciences Fondamentales et Biomédicales, Université de Paris, Paris, France; ^2^Departamento de Neurofisiología Celular y Molecular, Instituto de Investigaciones Biológicas Clemente Estable, Montevideo, Uruguay

**Keywords:** analog – digital signaling, antidromic, axon, neuron, action potential, subthreshold

## Abstract

Analog signaling describes the use of graded voltage changes as signals in the axonal compartment. Analog signaling has been described originally in invertebrates but more recent work has established its presence in the mammalian brain (Alle and Geiger, 2006; Shu et al., 2006). In recent years, many different groups have contributed to the understanding of the physiological significance of analog signaling from a cellular perspective (for a recent review the reader may take a look at the work by Zbili and Debanne, 2019 in this *Frontiers in Neuroscience* Special Issue). The great majority of the experimental work related to analog signaling, however, concerns the propagation of subthreshold voltage changes from the soma to the axon. Much less attention has been paid to the propagation of subthreshold voltage changes in the opposite direction, from the axon to the soma, or to the propagation of local signals within the axon. In this mini review we will describe these other variants of analog signaling that we call here “antidromic” coupling and “local” coupling.

## Introduction

The term “analog signaling” is used to describe the passive propagation of electrical signals among different neuronal compartments. In recent years it became clear that subthreshold or passive (analog) coupling between the somatodendritic and the axonal compartments is far more prevalent than what was expected 15 years ago (in invertebrate neurons the existence of analog signaling has been recognized a long time ago, Marder, 2006; Zbili and Debanne, 2019). This is probably due, at least in part, to the technical improvements that have been accomplished by different labs around the world, which allow to perform electrophysiological recordings and high-resolution imaging from single axonal varicosities.

Although there are some examples in the literature showing that some synapses can operate on the sub-threshold regime (i.e., in a graded manner, like in the vertebrate photoreceptor, Heidelberger, 2007), the majority of synapses operate in a digital fashion, where release happens when the action potential (AP) reaches the presynaptic varicosity. Nevertheless, the value of the local potential at the release site before the AP can influence the amount of release. This has led to the term “Analog–Digital” transmission, to stress the fact that the membrane potential at the varicosity right before the arrival of a spike can affect the AP shape and hence, release (see section on “Intra-Axonal Analog Signaling”).

**FIGURE 1:**
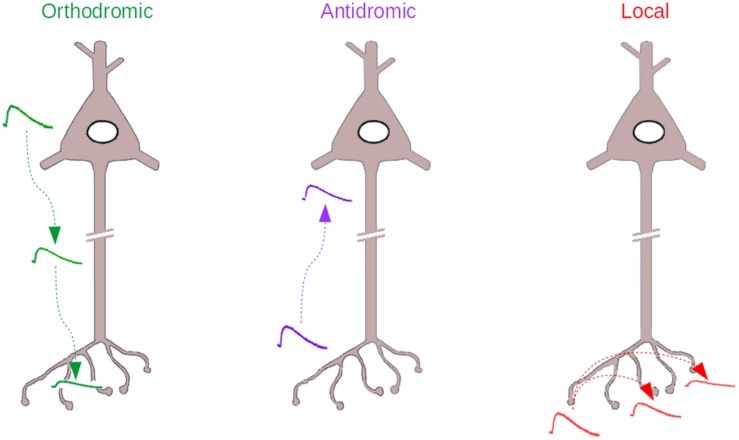
The three types of Analog signaling that have been described in the literature. *Left*, the orthodromic signaling, where somatodendritic voltage changes propagate to the axon. *Middle*, the antidromic signaling, where axonal voltage changes propagate backward to the axon initial segment and soma. *Right*, the local signaling, where axonal voltage changes propagate within the axon, to other varicosities, without escaping from the axonal compartment.

Even more challenging than this orthodromic analog coupling (Figure 1, *Left*), is the idea that the axonal compartment may also be the input compartment of a neuron and that some of the signals that originate in the axon may reach the soma and affect neuronal excitability (Figure 1, *Middle*). This idea challenges Ramón y Cajal’s dynamic polarization theory, that states that information in the nervous system is unidirectional, flowing from the dendrites to the soma to the axon. In antidromic signaling, the signal goes on the contrary from the axon to the soma. The present review discusses this paradoxical, often neglected form of axonal signaling, as well as another form of analog signaling that can be considered a variant of the previous one, “local” signaling, where axonal voltage changes remain confined to the axonal compartment (Figure 1, *Right*).

## The Axonal Length Constant, “λ”

Transmission of electrical signals in the axon can be approached by approximating the axon structure with a cable. The extent of propagation of passive signals in a cable of infinite length at steady state follows an exponential function of distance. The length constant (lambda, “λ”) depends on the cable internal resistance (the cytoplasmic resistance, which is a function of the cable diameter) and on the membrane resistance of the axon (Rall, 1969a,b; Rall and Rinzel, 1973).

While the value of lambda does not depend on the direction of the signal propagation for an infinite cable, directionality becomes important for finite length axon cables linked to other axonal or somatic compartments. Specifically, the voltage changes produced either in the soma or in the axon by some current injected in the other compartment will depend on the passive characteristics of the target compartment (the soma when the current is injected in the axon and the axon when the current is injected in the soma), that is, input resistance and membrane capacitance. This was recently studied in detail by Hu and Bean (2018), who made simultaneous patch-clamp recordings from the soma and the axon of cortical pyramidal neurons and quantified the degree of coupling in both directions, ortho and antidromic. In their study, the authors showed that coupling is stronger in the orthodromic than in the antidromic direction, and they attributed this effect to the impedance mismatch between the two compartments: the input resistance being much lower in the soma, an axonally injected current will induce a strong local (i.e., axonal) voltage change and a much smaller somatic voltage change. As pointed out by Thome et al. (2018), who showed a similar asymmetric coupling between the axonal and the somatic compartment of hippocampal pyramidal neurons, the input capacitance of each compartment will also have a kinetic effect: the higher the capacitance (soma), the slower will be the voltage change; the smaller the capacitance (axon), the quicker the voltage change will be.

In their study, Hu and Bean (2018) also showed that the axonal and somatic resting membrane potentials differ by a few millivolts due to the differential expression of different voltage dependent conductances. In the neurons under study, the somatodendritic Ih current strongly influences the axonal membrane potential (blocking Ih equalizes the somatic and the axonal Vm), but the axonal KV7 current has a negligible effect on the somatodendritic potential. This, again, can be explained by the asymmetry between orthodromic and antidromic coupling.

Although asymmetric coupling may be a general feature of mammalian neurons, its extent is expected to vary markedly depending on cell size. In smaller and more compact cells, like GABAergic interneurons or cerebellar granule cells, where the somatic input resistance is much higher, the asymmetry may be expected to be smaller than in big cells (like the cortical pyramidal cells studied by Hu and Bean, 2018). In these cells, one can expect a much bigger influence of axonal activity on the somatic membrane potential. The exact contribution of the size and general morphological features of a cell on the impact of the orthodromic coupling on cellular excitability remains to be characterized. In the next section we will describe some examples.

## Antidromic Signals Mediated by Axonal Ionotropic Receptors

Axonal, or presynaptic ionotropic receptors have been described in a large variety of neuronal types in the central and peripheral nervous system (MacDermott et al., 1999). The best documented presynaptic receptors are probably GABA_A_ receptors (GABA_A_Rs), which have been originally described in sensory primary afferents of the spinal cord (for a recent review on axonal GABA_A_Rs please see Trigo et al., 2008). In this preparation, GABA is released from the presynaptic compartment at axo-axonic synapses, and activation of presynaptic GABA_A_Rs results in presynaptic inhibition (a decrease in the amount of neurotransmiter released from the presynaptic side).

Following the pioneering work of Frank and Fuortes (1957), axonal GABA_A_Rs have been found in multiple neuronal cell types, and their mode of action has been studied in detail. In some cases activation of axonal GABA_A_ receptors appeared to be transmitted antidromically to the soma. An early example is a study by Pouzat and Marty (1999), who described an autoreceptor GABA_A_ current recorded from the soma of cerebellar molecular layer interneurons (MLIs, stellate and basket cells, parvalbumin positive GABAergic interneurons of the cerebellar cortex) that depends on the released GABA binding back to GABA_A_Rs located on the releasing cell (so-called axonal GABA_A_ autoRs). It was shown later by Mejia-Gervacio and Marty (2006) that GABA_A_ autoRs can shape MLI firing. In 2010 we extended this work and showed that in MLIs the activation of GABA_A_Rs in a single varicosity generates a measurable signal (a “premini,” from “presynaptic miniature” current) that can back-propagate to the soma. More recently, we used calcium photolysis in single varicosities to demonstrate that the voltage changes associated with preminis are able to change the cell excitability, probably by modifying AP threshold in the AIS (Zorrilla de San Martin et al., 2015).

Interestingly, the voltage changes produced by the activation of axonal GABA_A_Rs in MLIs are shaped by the activation of voltage-dependent conductances in the axon. In the work by Mejia-Gervacio and Marty (2006), the authors showed that the effects of the autoreceptor-mediated responses on cellular excitability are counterbalanced by the Ih conductance (a cationic conductance that has a depolarized reversal potential and that is activated by membrane potential hyperpolarization; in MLIs, this conductance is located primarily in the axon, Southan et al., 2000; Luján et al., 2005). More recently, we showed that autoR-mediated depolarizations are amplified by a voltage-dependent sodium conductance, probably a persistent sodium current (Zorrilla de San Martin et al., 2015). These experiments highlight the complex interactions that exist between different ligand and voltage-dependent conductances in the axon and stress the necessity to perform direct recordings from the axonal compartment in order to get a clear understanding of axonal physiology.

Cerebellar granule cells constitute another cell type where signals originating in the axon can reach the soma and change the cell excitability. It was first shown in Stell et al. (2007) that granule cell axons, known as parallel fibers, possess GABA_A_Rs; their activation increases GABA release, which implies a local depolarizing effect. More recently it was shown that these local, GABA_A_-mediated depolarizations can also reach the somatic compartment and change granule cell excitability (Pugh and Jahr, 2011, 2013; Stell, 2011; Dellal et al., 2012), including by directly inducing spiking in the axon (Pugh and Jahr, 2011) (see section below on “Ectopic APs,” EAPs).

In their study, Dellal et al. (2012) provide some details on the mechanism by which axonal GABA_A_Rs increase granule cell’s excitability. By performing a computational model of the cell, the authors show that the increases in local, axonal excitability and those in somatic excitability produced by the antidromic propagation of subthreshold signals are exquisitely sensitive to the exact chloride equilibrium potential. The increase in conduction velocity observed experimentally, on the other hand, is very sensitive to the voltage dependence of sodium channel inactivation, again highlighting the critical role that axonal voltage-dependent conductances have for the behavior of the axon (Dellal et al., 2012).

## Antidromic Signals Mediated by Voltage-Dependent Conductances

It is well established that the axonal compartment has a specific population of voltage-dependent channels that differs from that of the somatodendritic compartment. These channels contribute to axonal AP propagation and fidelity (see, for example, Hu and Jonas, 2014) and determine the exact AP waveform (see, for example, Geiger and Jonas, 2000) and hence, transmitter release. In the Calyx of Held, a giant synaptic terminal of the auditory pathway in the brainstem, different stimulation paradigms produce varying AP trains, with a typical afterhyperpolarization (due to a Na^+^/K^+^ ATPase pump; Kim et al., 2007) and afterdepolarization (sensitive to riluzole, presumably produced by a persistent type of Na^+^ current; Paradiso and Wu, 2009). By combining direct terminal recordings from the Calyx and extracellular stimulation of the parent axon, Paradiso and Wu (2009) demonstrated that the axonal voltage changes (produced locally in the Calyx) can travel back to the soma for hundreds of microns and affect AP threshold. The exact effect on excitability of the axonal voltage changes described by Paradiso and Wu (either and increase or a decrease in AP probability) depended on the exact spiking pattern, with the afterdepolarization dominating at the beginning of the burst and the afterhyperpolarization taking over at later stages.

The experiments performed by Paradiso and Wu show that the common view that somatodendritic voltage-dependent conductances determine spiking is an oversimplification. These experiments emphasize the role of the axon in regulating the spiking pattern of the neuron, which is extremely relevant for the physiology of the neuron because it controls the characteristics of synaptic release and therefore, plasticity mechanisms.

## Intra-Axonal Analog Signaling

Local or “intra-axonal” analog signaling has been difficult to study for technical reasons: demonstration of this type of coupling requires recording from 2 varicosities simultaneously. To perform such a challenging experiment, it is necessary to select a special preparation such as the cerebellar primary culture preparation (Kawaguchi and Hirano, 2013; Kawaguchi and Sakaba, 2015) where the axon is planar and where individual cells can be transfected with fluorescent markers, greatly facilitating the identification of the varicosities to be patched. Zorrilla de San Martin et al. (2017) recorded from 2 Purkinje cell varicosities simultaneously. When some current was injected in one varicosity it produced a local voltage change and a voltage change in the neighboring varicosity as well. The voltage change decremented exponentially as a function of distance along the axon, as can be expected from a passive phenomenon, with a λ close to 100 μm.

What kind of signal could give rise to local axonal depolarization in Purkinje cells? In their paper, Zorrilla de San Martín et al. found that Purkinje cells boutons express GABA_A_ receptors. In several neuron types the reversal potential for GABA_A_ receptors, E_GABA_, has been found to be less negative in the axon than in the soma (Price and Trussell, 2006; Szabadics et al., 2006). Purkinje cell axons are no exception. By performing perforated, cell-attached recordings, it was shown that axonal receptors mediate a response that has a reversal potential around −47 mV, more depolarized than the somatic E_GABA_ (∼−73 mV). Therefore, local activation of axonal GABA_A_ receptors in Purkinje cell axons induces a depolarizing signal that is propagated to neighboring varicosities. The upstream signal seems to be axonal firing and subsequent GABA_A_ autoR activation (like in cerebellar interneurons), but other options such as spillover GABA release from neighboring axons, or GABA release from glia, are also possible. Concerning this last possibility, evidence in other systems indicates that the original signal may come from non-neuronal cells, like astrocytes. Sasaki et al. (2011) showed that glutamate released by astrocytes surrounding the axonal varicosities of CA3 cells can broaden the AP. Although not shown by the authors, this effect may implicate depolarization propagating across varicosities.

Here, it is important to stress the fact that the intra-axonal, local voltage changes, although sub-threshold, can affect release by modulating the availability of voltage-dependent channels in the varicosity and therefore, the shape of the incoming AP. Also, sub-threshold local voltage changes may also affect release without inducing any change in AP shape, for example by modulating the basal calcium levels or the availability of voltage-dependent calcium channels just before release. For an extensive list of references on this issue the reader can consult the recent review by Zbili and Debanne (2019) and notably their Table 1.

While local analog signaling in the axon domain remains scarcely documented, it is probably even more common than the two other forms of axonal analog signaling, ortho- and antidromic signaling. If some axonal signals reach the soma, as previously discussed, then they must also reach other varicosities, simply because the intervaricosity distance is smaller than the varicosity to soma distance. In the case of long-projecting neurons, such as cortical pyramidal neurons or spinal cord motoneurons, where the axonal length reaches tens and sometimes hundreds of millimeters, local, subthresold axonal signals may hardly ever reach the soma but may have a strong influence on neighboring varicosities. In such a case, the axonal compartment may largely operate independently from the soma, opening new and unexpected possibilities for neuronal computation.

## The Case of Ectopic Activity

So far, we have considered coupling between different axonal compartments for voltage signals in the subthreshold regime. However, it is important to appreciate the fact that local events of high amplitude may reach threshold and evoke spiking activity directly in the distal axon. These locally generated action potentials, called EAPs, differ radically from normal APs that are generated in the axon initial segment. They violate Cajal’s polarization rule and travel backward to the soma. EAPs have been extensively documented in invertebrates (for an excellent review of EAPs see Pinault, 1995). In recent years, a few groups have shown conclusively the existence of ectopic spikes in the normal and the physiopathological context in mammals. We will highlight here some of the former examples.

Sheffield et al. (2011) showed that repeated current injection into hippocampal interneurons eventually produces persistent firing, a form of spiking that outlasts the current injection for tens of seconds. The authors showed that persistent firing is not initiated in the soma but in the distal axon and is blocked by gap junction blockers (electrical coupling through axonal electrical synapses has already been shown by Schmitz et al., 2001), although it is still present in connexin 36 knockouts (Sheffield et al., 2013). Interestingly, persistent firing can spread from the stimulated cell to a neighboring, non-stimulated cell.

During network oscillations in the hippocampus (*in vitro* gamma frequency), EAPs are generated in the axon of CA3 pyramidal cells but fail to reach the soma (Dugladze et al., 2012) (giving rise to the so-called “spikelets”); according to the author’s results, this is because the voltage changes are shunted by axo-axonic GABAergic synapses on the axon initial segment. Surprisingly, axo-axonic cells are more efficient in preventing the back-propagated EAPs to reach the soma than in preventing the generation of orthodromic spikes (which is usually the main function attributed to axo-axonic cells). Spikelets have also been described in CA1 pyramidal cells *in vivo*, where they represent around one third of the spiking activity of the neuron (Epsztein et al., 2010). Interestingly, the spikelet frequency is modulated according to the location of the animal in space, indicating that spikelets play an important role in spatial exploration.

In a recent paper, Thome et al. (2018) showed that EAPs (probably the spikelets in Epsztein’s work) can be triggered in hippocampal CA1 pyramidal neurons by axonal stimulation. In their paper, the authors showed that EAPs reliably propagate toward the soma, and further showed that their occurrence can be modulated by subthreshold synaptic activity, either depolarizing or hyperpolarizing, in the somatodendritic compartment. With this paper Thome and collaborators nicely integrated some of the concepts that have been discussed in this review: the analog, ortho and antidromic coupling between the somatodendritic and axonal compartments of CA1 pyramidal neurons is very prominent; in some special occasions, which remain to be exactly determined, the axon may be capable of generating EAPs that reach the soma and whose activity is, at the same time, controlled by somatodendritic activity.

## Conclusion and Future Directions

All the experimental data that has been collected during the past two decades or so has progressively changed our view of the physiology of the axonal compartment. Clearly, the axon cannot be considered as a simple transmission cable, and it rather emerges as a complex computational unit that increases the operational capabilities of the neuron (Debanne et al., 2011). Today, it is generally accepted that sub threshold voltage changes originating primarily in the dendrites can passively propagate to the axon, where they modify voltage-dependent conductances and AP shape. In this mini-review we summarized recent work showing that signals originating primarily in the axon can back-propagate to the soma, giving rise to a type of antidromic (and sometimes local) analog signaling. For technical reasons, the pace of research in the field of axon signaling has been relatively slow up to now. However, we predict that the development of new, brighter and faster, genetically encoded voltage indicators (for a recent review see Panzera and Hoppa, 2019) will soon open new avenues in the study of the axon, notably by allowing the simultaneous imaging of different axonal regions, far away from the somatic compartment of neurons.

## Author Contributions

FT wrote the manuscript and prepared the figure.

## Conflict of Interest Statement

The author declares that the research was conducted in the absence of any commercial or financial relationships that could be construed as a potential conflict of interest.
